# Transcriptomic profiling of secukinumab-treated psoriatic arthritis reveals potential novel response-associated pathways

**DOI:** 10.1093/rheumatology/keag193

**Published:** 2026-04-15

**Authors:** Nahdia Afiifah Abdul Jalil, Hector Chinoy, Meghna Jani, Anne Barton, Charlotte Thompson, Paul Martin, Nisha Nair, James Bluett, for the Outcomes of Treatment in Psoriatic Arthritis Study Syndicate (OUTPASS), James Bluett, E G Chelliah, C Chattopadhyay, P Ho, A Barton, M Castelino, I Bruce, R Gorodkin, K Hyrich, B Parker, H Chinoy, T O’Neil, A Herrick, A Jones, R Cooper, W Dixon, B Harrison, M Jani, A Low, E Korendowych, N McHugh, W Tillett, N Goodson, S Lane, L Shand, I Pande, I Gaywood, F Rees, M Rutter, S Hayat, J F McHale, A C Jones, P Lanyon, A Gupta, P A Courtney, A Srikanth, A Abhishek, S Kyle, R Manhas, A Nandagudi, S Selvan, A Bharadwaj, N Gendi, R Alshakh, S Naz, M Ahmad, L Das, M Pattrick, A P Bowden, E E Smith, P Klimiuk, D J Speden, M Bukhari, S Kavaklieva, L Ottewell, M Massarotti, J Packham, P Watson, P Sanders, S Haque, B Pal, E Bruce, Z Karim, K Mackay, H Shiels, J Taylor, R Jeffery, P Nandi, C Filer, A Ismail, L Mercer, A Hassan, A Russell, M Durrani, W Hassan, A Samanta, P Sheldon, J Francis, A Kinder, R Neame, A Moorthy, M Bombardieri, S Kelly, J Maxwell, M Akil, S Till, L Dunkley, R Tattersall, R Kilding, T Tait, K-P Kuet, B Grant, M Kazmi, D Graham, V E Abernethy, A R Clewes, J K Dawson, S Siebert, G Fragoulis, D Mewar, E J Tunn, K Nelson, T D Kennedy, C Dubois, K Douglas, E Ladoyanni, C Koutsianas, N Erb, R Klocke, A J Whallett, A Pace, R Sandhu, H John, S A Young Min, A Cooper, J M Ledingham, R G Hull, F McCrae, Wong, Shaban, K Putchakayala, R Kumari, G Smith, C Marguerie, P Reynolds, C Thornton, C Gorman, C Murphy, D Roy, S Horton, M Castelino

**Affiliations:** Versus Arthritis Centre for Genetics and Genomics, Centre for Musculoskeletal Research, Manchester Academic Health Science Centre, University of Manchester, Manchester, UK; Department of Anatomy, Faculty of Medicine, Universiti Kebangsaan Malaysia, Kuala Lumpur, Malaysia; Versus Arthritis Centre for Genetics and Genomics, Centre for Musculoskeletal Research, Manchester Academic Health Science Centre, University of Manchester, Manchester, UK; NIHR Manchester Biomedical Research Centre, Manchester University NHS Foundation Trust, Manchester Academic Health Science Centre, Manchester, UK; NIHR Manchester Biomedical Research Centre, Manchester University NHS Foundation Trust, Manchester Academic Health Science Centre, Manchester, UK; Centre for Epidemiology Versus Arthritis, Centre for Musculoskeletal Research, University of Manchester, Manchester, UK; Versus Arthritis Centre for Genetics and Genomics, Centre for Musculoskeletal Research, Manchester Academic Health Science Centre, University of Manchester, Manchester, UK; NIHR Manchester Biomedical Research Centre, Manchester University NHS Foundation Trust, Manchester Academic Health Science Centre, Manchester, UK; University Hospital Sussex NHS Foundation Trust, UK; Versus Arthritis Centre for Genetics and Genomics, Centre for Musculoskeletal Research, Manchester Academic Health Science Centre, University of Manchester, Manchester, UK; NIHR Manchester Biomedical Research Centre, Manchester University NHS Foundation Trust, Manchester Academic Health Science Centre, Manchester, UK; Versus Arthritis Centre for Genetics and Genomics, Centre for Musculoskeletal Research, Manchester Academic Health Science Centre, University of Manchester, Manchester, UK; NIHR Manchester Biomedical Research Centre, Manchester University NHS Foundation Trust, Manchester Academic Health Science Centre, Manchester, UK; Versus Arthritis Centre for Genetics and Genomics, Centre for Musculoskeletal Research, Manchester Academic Health Science Centre, University of Manchester, Manchester, UK; NIHR Manchester Biomedical Research Centre, Manchester University NHS Foundation Trust, Manchester Academic Health Science Centre, Manchester, UK

**Keywords:** precision medicine, predictive biomarkers, psoriatic arthritis, transcriptomics, molecular signatures

## Abstract

**Objectives:**

IL-17A inhibitors are therapeutic options in PsA, but response is not universal. Evidence from other inflammatory arthritides suggests differential gene expression may predict the outcomes. This study aimed to identify transcriptomic predictive biomarkers of response in PsA patients commencing secukinumab.

**Methods:**

Participants were recruited to OUtcomes of Treatment in Psoriatic Arthritis Study Syndicate (OUTPASS), a prospective observational cohort study of patients with PsA initiating advanced therapeutics. Samples for this analysis were chosen based on extreme phenotype response. Whole-blood RNA sequencing was performed longitudinally in 13 secukinumab-treated patients at baseline (pre-treatment) and at 3 months post-treatment, with response evaluated at 3 months using the DAS28 criteria and the Psoriatic Arthritis Response Criteria. Differential gene expression analysis, Ingenuity Pathway Analysis (IPA), and weighted gene co-expression network analysis (WGCNA) were performed to identify significantly differentially expressed genes (DEGs) (adjusted *P* < 0.05, |log_2_ fold change| ≥ 1), enriched pathways (*P* < 0.05), and co-expressed gene modules. Immune cell subset proportions were estimated by deconvolution, and hub genes were identified by integrating DEGs and WGCNA, with overlapping genes defined as potential driver genes.

**Results:**

*IGHV3-64D* and *IGHV1-46* were differentially expressed at baseline, 3 months, and sustained over time in the responder group (adjusted *P *< 0.05). Five overlapping genes (*GMPR*, *CDC34*, *DMTN*, *UBXN6* and *SLC25A39*) were identified as potential drivers. Functional analysis indicated a potential contribution of metabolic pathways to the modulation of therapeutic response.

**Conclusion:**

We identified two genes as pre-treatment predictive biomarkers of secukinumab response that persisted over time. Integration with WGCNA revealed five additional candidate genes. These genes are implicated in metabolic pathways, which may modulate the secukinumab response. These findings warrant further validation.

Rheumatology key messages

- *IGHV3-64D* and *IGHV1-46* showed significant and sustained expression at baseline and at 3 months post-secukinumab treatment.
- Both genes are associated with B cell–mediated immunity, suggesting their potential role in PsA.
- Metabolic pathways may play a role in influencing the therapeutic response to secukinumab.

## Introduction

PsA is a clinically and molecularly heterogeneous disease, with diverse inflammatory and immune-mediated manifestations reflecting variability in its underlying pathogenic mechanisms and genetic origins. To address this complexity, a wide range of targeted therapies have been developed to inhibit specific immune cells, cytokines, and molecules [1]. Among these, medications targeting the pro-inflammatory cytokine IL-17A, such as secukinumab and ixekizumab, have demonstrated clinical efficacy consistent with pathogenic involvement of IL-17A in PsA [2].

IL-17A signalling, mediated by CD8^+^ and CD4^+^ T cells, plays a key role in PsA pathogenesis [2]. Additionally, *IL-17A* and *IL-17F* gene polymorphisms have been shown to be associated with PsA [2]. Type 3 innate lymphoid cells (ILC3s) are a major source of IL-17A and are abundantly found in the synovium of patients with PsA, where their levels correlate with PsA disease activity [3]. Furthermore, entheseal-resident IL-17A–producing cells, including ILC3s and γδ T cells, have been identified as potential local drivers of enthesitis [4, 5]. Activation of the receptor activator of nuclear factor kappa-light-chain-enhancer of activated B cells (NF-κB) ligand (RANKL) promotes bone resorption and consequent joint deformity in PsA by triggering differentiation of osteoclast precursor cells into activated osteoclasts [6].

Although secukinumab has been reported to achieve up to 80% efficacy based on Minimal Disease Activity criteria, ∼20% of patients discontinue secukinumab therapy due to reduced effectiveness [7, 8]. In such cases, disease progression may lead to structural joint damage, pain and disability, and reduced work productivity and quality of life [9, 10]. These limitations underscore the need for reliable molecular biomarkers to guide timely intervention and inform the choice of therapy. The relationship between genetics and clinical phenotypes can be explored through gene expression profiling combined with pathway enrichment and functional analysis. Supporting this approach, previous transcriptomic studies of CD4^+^ T cells from patients with PsA treated with biologics have identified the Rho-GTPase pathway and its key genes, *RAC1* and *ROCK2*, as potential regulators of treatment response, suggesting a promising avenue for precision medicine in PsA [11].

In this study, we analysed whole blood RNA sequencing data from PsA patients treated with secukinumab to characterize transcriptomic signatures of therapeutic response, with a focus on identifying pretreatment transcriptomic-based biomarkers predictive of therapeutic response and enriched pathways related to the dynamics of therapeutic response.

## Materials and methods

### Experimental design and study participants

The participants of this study were PsA patients enrolled in the prospective observational UK multicentre study for OUtcomes of Treatment in Psoriatic Arthritis Study Syndicate (OUTPASS), described elsewhere [12, 13]. The inclusion criteria for this study were: (i) fulfilment of the Classification Criteria for Psoriatic Arthritis (CASPAR) or diagnosis of PsA by a clinician; (ii) initiation of a biologic or small-molecule therapy; (iii) age ≥ 18 years; and (iv) availability of baseline PsA response criteria (PsARC) or 28-joint DAS (DAS28) data. To be eligible for the current analysis, patients were required to be prescribed secukinumab, having available data for baseline (pre-treatment) and for the 3-month PsARC and DAS28. Where data was available, secukinumab was administered at a dose of 150 or 300 mg monthly. One participant was recorded as receiving 300 mg/week at the baseline time point, which may represent the initial loading dose. Samples were selected using an extreme phenotype approach, based on disease activity measures. Responders were defined as individuals who achieved all of the following criteria: a 3-month PsARC response, a DAS28 score of <3.2 at the 3-month timepoint, and who ranked among those participants having the greatest change in DAS28 score within the cohort. In contrast, non-responders were classified as those with the lowest (or negative) changes in DAS28 score.

Data collection for each patient included: age, sex, disease duration (defined as the time from year of diagnosis to the start of biologic therapy), BMI, smoking status, concurrent use of conventional synthetic DMARDs (csDMARDs), NSAIDs, and clinical patterns of the disease. Changes in the DAS28 score and PsARC from baseline to 3 months were used as outcome measures.

### RNA isolation and sequencing

Whole blood was collected in Tempus™ Blood RNA Tubes (Thermo Fisher Scientific) at baseline (pre-secukinumab) and at 3 months and stored at −80°C prior to RNA extraction. Total RNA was extracted using *MagMAX^TM^* (Thermo Fisher Scientific), followed by globin mRNA depletion using the *GLOBINclear^TM^* kit (Thermo Fisher Scientific). Quality and integrity of the RNA samples were assessed using a 4200 TapeStation (Agilent Technologies). Libraries were generated using the *Illumina^®^ Stranded mRNA Prep. Ligation* kit (Illumina, Inc.) according to the manufacturer’s protocol. Paired-end sequencing was performed on the Illumina NovaSeq 6000 platform.

### Gene expression and pathway analysis

Raw RNA sequencing reads in FASTQ format were subjected to initial quality control using FastQC [14] to assess sequencing quality metrics. Adapter sequences and low-quality bases were trimmed using Trimmomatic [15]. Cleaned reads were then aligned against the human reference genome (hg38) using the STAR aligner [16]. Post-alignment processing included duplicate marking with Picard’s *MarkDuplicates* and computation of alignment metrics using *CollectRnaSeqMetrics* to evaluate mapping quality and coverage [17]. Read quantification was performed with *featureCounts*, assigning aligned reads to exons grouped by gene in the GENCODE v45 annotation [18, 19]. Raw counts were corrected to remove the hidden batch effect using the *RUVseq* package in R [20]. Differential expression analysis was conducted using DESeq2 [21], with models adjusted for age, sex and BMI. Genes with an absolute log_2_ fold change (|log_2_FC|) ≥ 1 and an adjusted *P*-value < 0.05 were considered significantly differentially expressed genes (DEGs). Data visualization was performed using the ggplot2, *enhancedVolcano* and *VennDiagram* R packages to generate heatmaps, volcano plots and Venn diagrams. Ingenuity Pathway Analysis (IPA) was performed to identify enriched pathways using all genes with *P* < 0.05 and log_2_FC ≥ 1, revealing the identification of differentially enriched pathways (*P *< 0.05) [22].

### Co-expression network analysis

We applied Weighted Gene Co-expression Network Analysis (WGCNA) to identify clusters of highly correlated genes, in our blood gene expression dataset [23]. Normalized expression data were analysed in R using the *blockwiseModules* function to construct a signed topological overlap matrix (TOM) and identify co-expression modules. Module eigengenes (MEs) were extracted, ordered, and correlated with clinical traits using Pearson correlation. The module most significantly associated with the clinical trait of interest was selected for further analysis, and its biological relevance was evaluated through Kyoto Encyclopedia of Genes and Genomes (KEGG) and Gene Ontology (GO) pathway enrichment analysis.

Hub genes within the key module were defined as those with high module membership (MM) MM > 0.8) and significant DEGs. Potential driver genes were identified as those showing overlap across these criteria and the top 30 hubs genes determined by the *cytoHubba* in Cytoscape based on the degree method [24, 25]. In parallel, intramodular connectivity (kWithin), which quantifies the strength of a gene’s connection within its module, was also computed to further support the hub gene identification.

### Deconvolution of gene expression profiles

CIBERSORTx, a reference-based deconvolution tool, was utilized to estimate the frequencies of immune cell subsets in whole-blood RNA-seq data [26]. Gene expression data derived from STAR were normalized to Transcripts Per Million (TPM). The LM22 signature matrix, which profiles 22 human hematopoietic immune cell subtypes, was selected as a reference [27]. Deconvolution was performed in absolute mode, with quantile normalization disabled and using 100 permutations. Verbose output was enabled for refined reporting and reproducibility.

### Patient and public involvement

Members of The Research User Network were involved throughout the research cycle of OUTPASS. Network members gave feedback for the patient-facing material and study design.

### Statistical analysis

The results are presented as mean with s.d. unless otherwise specified. Continuous variables were compared between responder groups at baseline and 3 months using Student’s *t* test or the Mann–Whitney *U* test, depending on the data distribution as assessed by normality testing. Categorical variables were analysed using Fisher’s exact test. A *P*-value of <0.05 indicated statistical significance. Statistical analyses were performed using STATA version 14 and R version 4.4.1.

## Results

### Patient demographics

A total of 20 patients (10 responders; 10 non-responders) were identified as fulfilling the eligibility criteria for this study, with 13 patients (7 responders; 6 non-responders) having both baseline and 3-month samples available. Table 1 shows the baseline demographics data and disease characteristics for both groups. There was no statistically significant difference in the baseline demographic variables between the two groups, although disease duration was notably higher in the non-responder group.

**Table 1:** Demographic and clinical characteristics of PsA patients.

	Responder (*n* = 7)	Non-responder (*n* = 6)	*P* value
Age, mean (s.d.)	62.7 (10.9)	62.2 (13.1)	0.94
Female, *n* (%)	5 (71.4%)	2 (23.8)	0.29
BMI, median (IQR)	28.1 (25.7–30)	30.4 (30.1–36.6)	0.12
Duration of disease, median (IQR)	4 (1–8)	14 (4–36)	0.11
Smoking, *n* (%)			0.49
* Never smoked*	1 (14.3%)	3 (50%)	
* Current smoker*	2 (28.6%)	1 (0.2%)	
* Ex-smoker*	4 (57.1%)	2 (0.3%)	
Doses of secukinumab			
* 150 mg, n (%)*	3 (42.9%)	2 (33.3%)	
* 300 mg, n (%)*	2 (28.6%)	3 (50%)	
csDMARDs, *n* (%)	3 (42.9%)	3 (50%)	1
Daily NSAIDs, *n* (%)	2 (28.6%)	2 (33.3%)	0.79
Extra-articular pattern			
* Nail-fold psoriasis*	5 (83.3%)	2 (33.3%)	0.24
* Enthesitis*	3 (50%)	3 (50%)	1
* Axial involvement*	0	4 (66.7%)	0.06
* Anterior uveitis*	0	1 (16.7%)	1
Baseline DAS28, mean (s.d.)	4.9 (1)	5.7 (1.2)	0.24
Baseline PsARC, mean (s.d.)			
* Swollen joint count*	5.2 (4.5)	8.8 (7.4)	0.32
* Tender joint count*	15.5 (9.2)	28 (24)	0.26
* Physician’s global assessment*	2.8 (0.4)	3.6 (1.1)	0.16
* Patient’s global assessment*	3.3 (1)	3.4 (1.5)	0.93
Change baseline to 3 months, mean (s.d.)			
DAS28	2.5 (0.5)	0.6 (0.6)	<0.01
PsARC			
* Swollen joint count*	4.3 (1.6)	1.8 (10)	0.58
* Tender joint count*	14.3 (9)	1.8 (9.5)	<0.05
* Physician’s global assessment*	1.6 (0.9)	0.2 (1.8)	0.16
* Patient’s global assessment*	2 (10)		<0.05

csDMARDs: conventional synthetic DMARDs; DAS28: 28-joint DAS; IQR: interquartile range; PsARC: PsA response criteria.

### Differential gene expression between the responder and non-responder groups across the time points, time-series analysis, and canonical pathways associated with the therapeutic response

**Figure 1:**
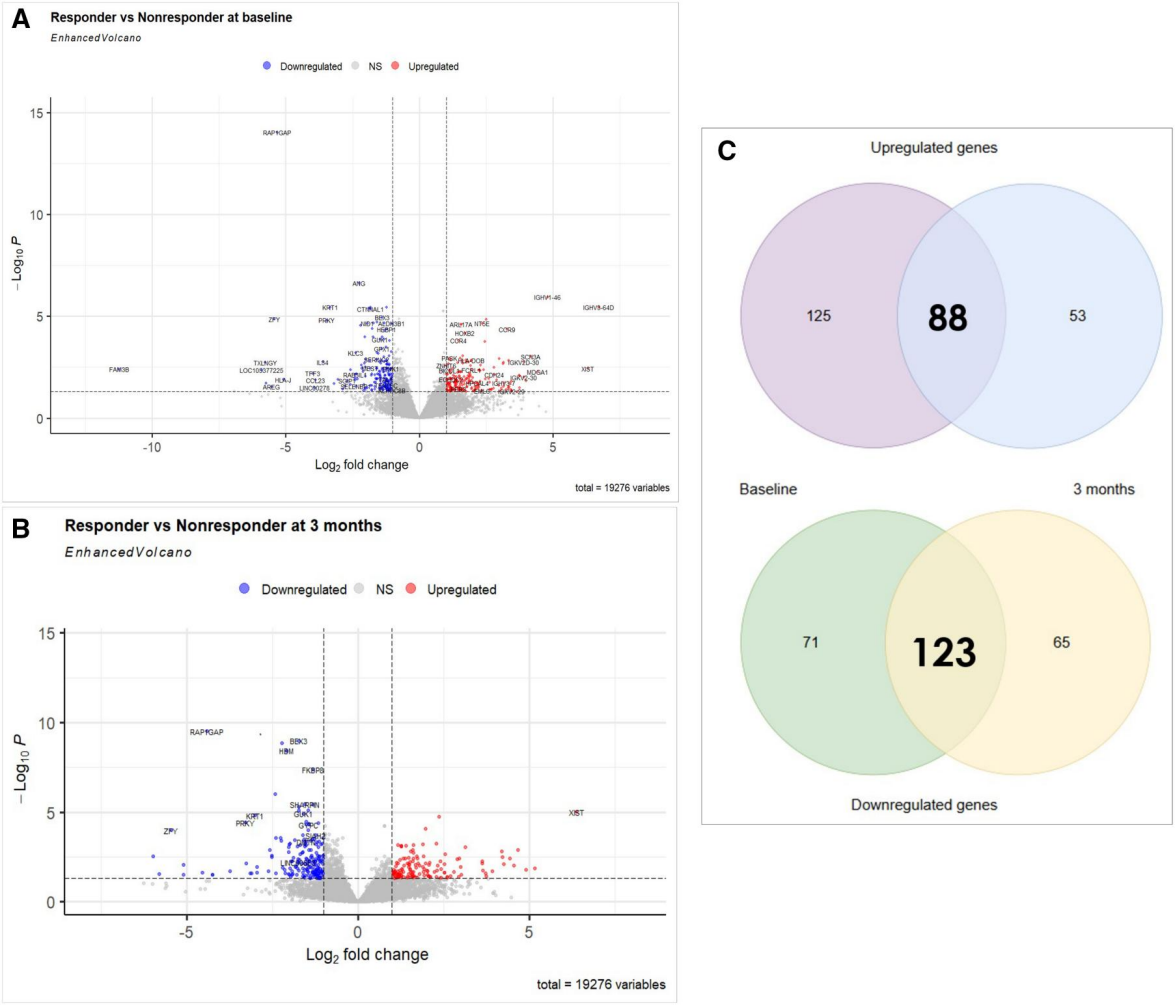
Enhanced volcano plot of differentially expressed genes between the responder and the non-responder groups (A) at baseline (B) after 3 months. Upregulated genes are located in the top-right quadrant, downregulated genes are in the top-left quadrant, and non-significant (NS) genes occupy lower and central portions of the plot. (C) Venn diagram illustrating the overlap of upregulated and downregulated genes at both time points between responder and non-responder groups

At baseline, we identified 407 DEGs between the responder and non-responder groups (Supplementary Table S1). Of these, at baseline, 194 genes were downregulated and 213 genes were upregulated in the responder group (Fig. 1A). At the 3-month time point, 325 genes were differentially expressed, with 184 downregulated and 141 upregulated genes in the responder group (Fig. 1B) (Supplementary Table S2). Additionally, we found 88 upregulated and 123 downregulated genes at both baseline and 3-month (Fig. 1C). Among the top five consistent DEGs are *IGHV3-64D, XIST, IGHV1-46, MDGA1* and *SCN3A* (Supplementary Table S3). Time-series analysis revealed that two of the top five DEGs, *IGHV3-64D* and *IGHV1-46*, remained significantly expressed over time (Supplementary Table S4).

**Figure 2:**
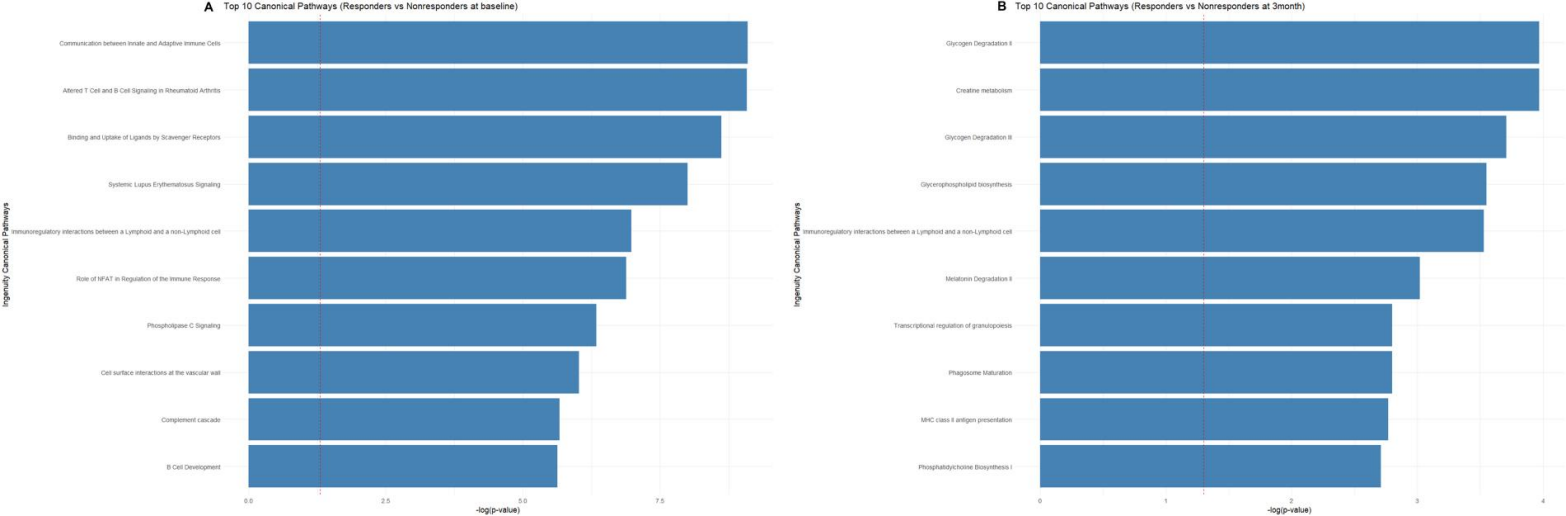
Bar plot of the top 10 canonical pathways of responder *vs* non-responder, ranked by –log(*P*-value). (A) at baseline and (B) after 3 months. The vertical dashed line indicates the threshold of statistical significance at –log(*P*-value) = 1.3 (equivalent to *P *< 0.05). NFAT = Nuclear factor of activated T-cells

The majority of the significantly enriched canonical pathways at baseline were associated with immune pathways, including communication between innate and adaptive immune cells, immunoregulatory interactions between a lymphoid and a non-lymphoid cell, altered T-cell and B-cell signalling in RA, and SLE signalling (Fig. 2A, Supplementary Table S5). Following 3 months of secukinumab treatment, IPA revealed a shift in pathway activity, with notable enrichment of pathways implicated in therapeutic response, including several belonging to the metabolic pathway group, specifically creatine metabolism, glycogen degradation, glycerophospholipid biosynthesis, and melatonin degradation (Fig. 2B) (Supplementary Table S6).

### Identification of key modules, functional analysis of key modules, and identification of potential hub genes

**Figure 3:**
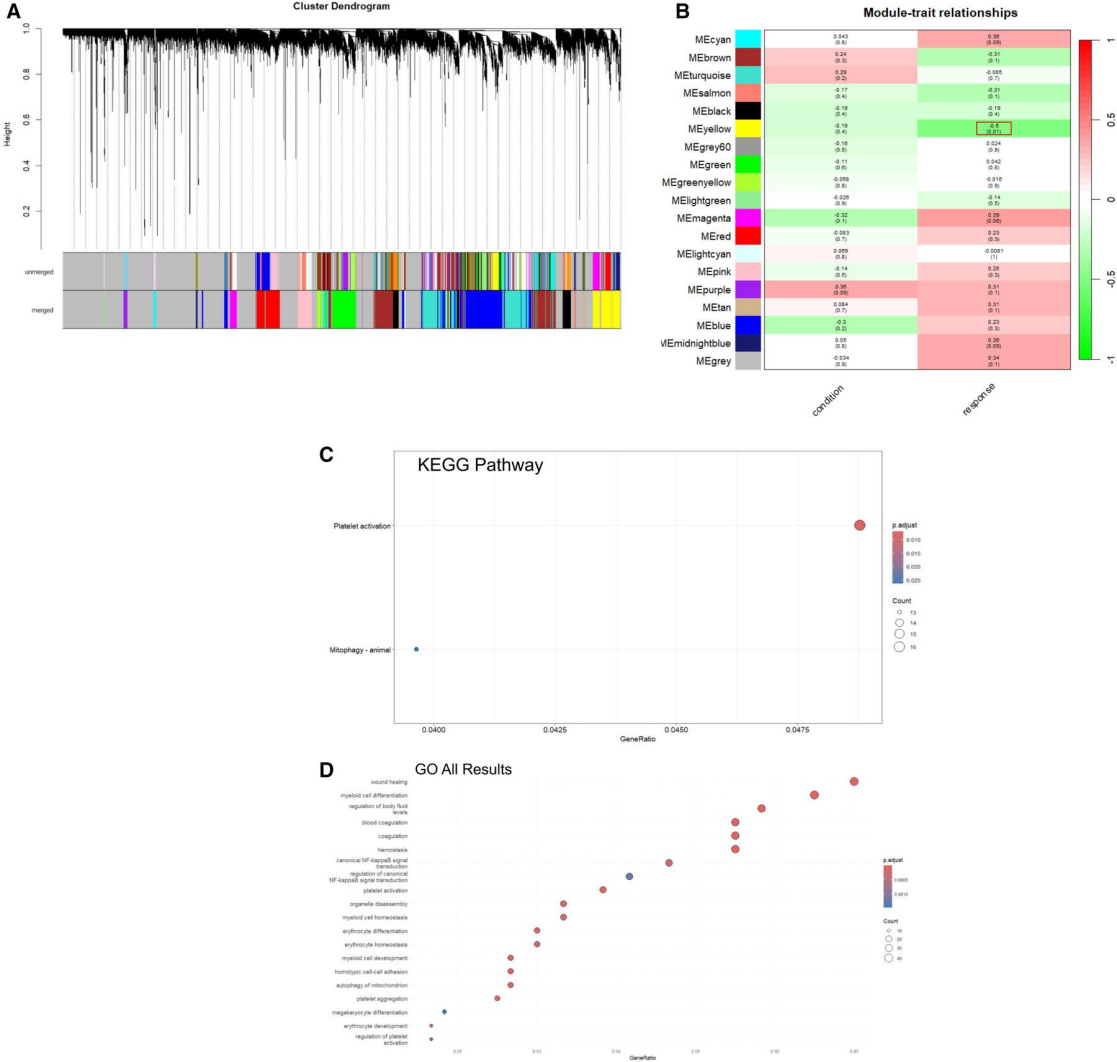
Cluster dendogram, module-trait associations of MEyellow module and pathway enrichment analysis related to treatment response. (A) The cluster dendrogram of module eigengenes. (B) Module–trait relationship. The MEyellow module showed a significant association with treatment response (*r* = –0.5, *P *= 0.01). Functional enrichment analysis for (C) Kyoto Encyclopedia of Genes and Genomes (KEGG) pathways and (D) Gene ontology (GO)

To further investigate the transcriptomic features of PsA in whole blood, we performed WGCNA. A cluster dendrogram was generated to visualize the hierarchical clustering of genes and to identify modules of co-expressed genes (Fig. 3A). Pearson correlation analysis of 12 modules with clinical traits (response and condition) revealed that the yellow module was negatively correlated with treatment response (*r* = −0.5, *P *= 0.01) (Fig. 3B). Functional enrichment analysis of genes in the yellow module using GO and KEGG is presented in Fig. 3C and D, respectively, with an adjusted *P*-value of <0.05 considered significant. GO analysis highlighted processes such as wound healing, focal adhesion, and blood coagulation, while KEGG revealed enrichment in platelet activation. Notably, platelet activation appeared in both KEGG and GO, suggesting a central role in the inflammatory response to treatment in PsA.

**Figure 4:**
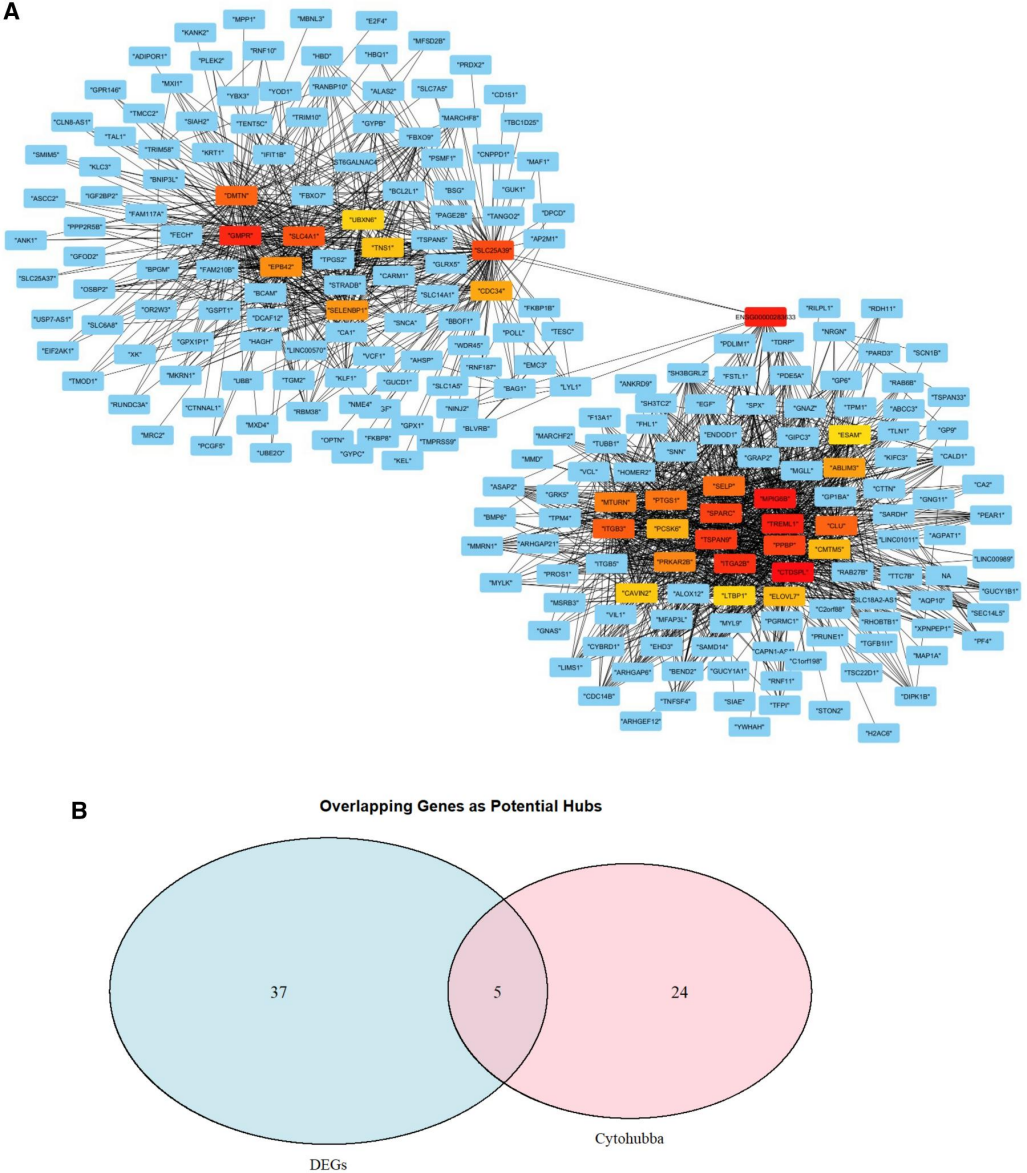
Common hub genes of the MEyellow identified by WGCNA and DEGs. (A) The top 75% of genes from the MEyellow were imported into Cytoscape to construct a co-expression network. Nodes represent genes, and edges represent protein–protein interactions. Thirty hub genes were identified using the degree method via the CytoHubba plugin. Node shading intensity reflects degree centrality, with the most intensely shaded nodes representing highly connected genes (top-ranking hubs) and lighter nodes indicating lower connectivity. (B) Venn diagram showing the overlap between genes in the co-expression network and DEGs, highlighting potential hub genes

The yellow module was comprised of 684 genes. Among these, 43 hub genes were identified based on set thresholds as having MM > 0.8 and being significant DEGs (Supplementary Table S7). To optimize computational efficiency, the top 75% of genes within the yellow module were imported and visualized using Cytoscape. Of 30 hub genes identified by the degree method in *cytoHubba* plug-in (Fig. 4A), 5 were considered potential hub genes (*GMPR, CDC34, DMTN, UBXN6* and *SLC25A39*) based on their overlap with both co-expression (MM > 0.8 and DEGs) and *cytoHubba* results (Fig. 4B). Consistently, intramodular connectivity analysis further supported *GMPR* as the central hub gene within its module.

### Immunoprofiling of peripheral blood

**Figure 5:**
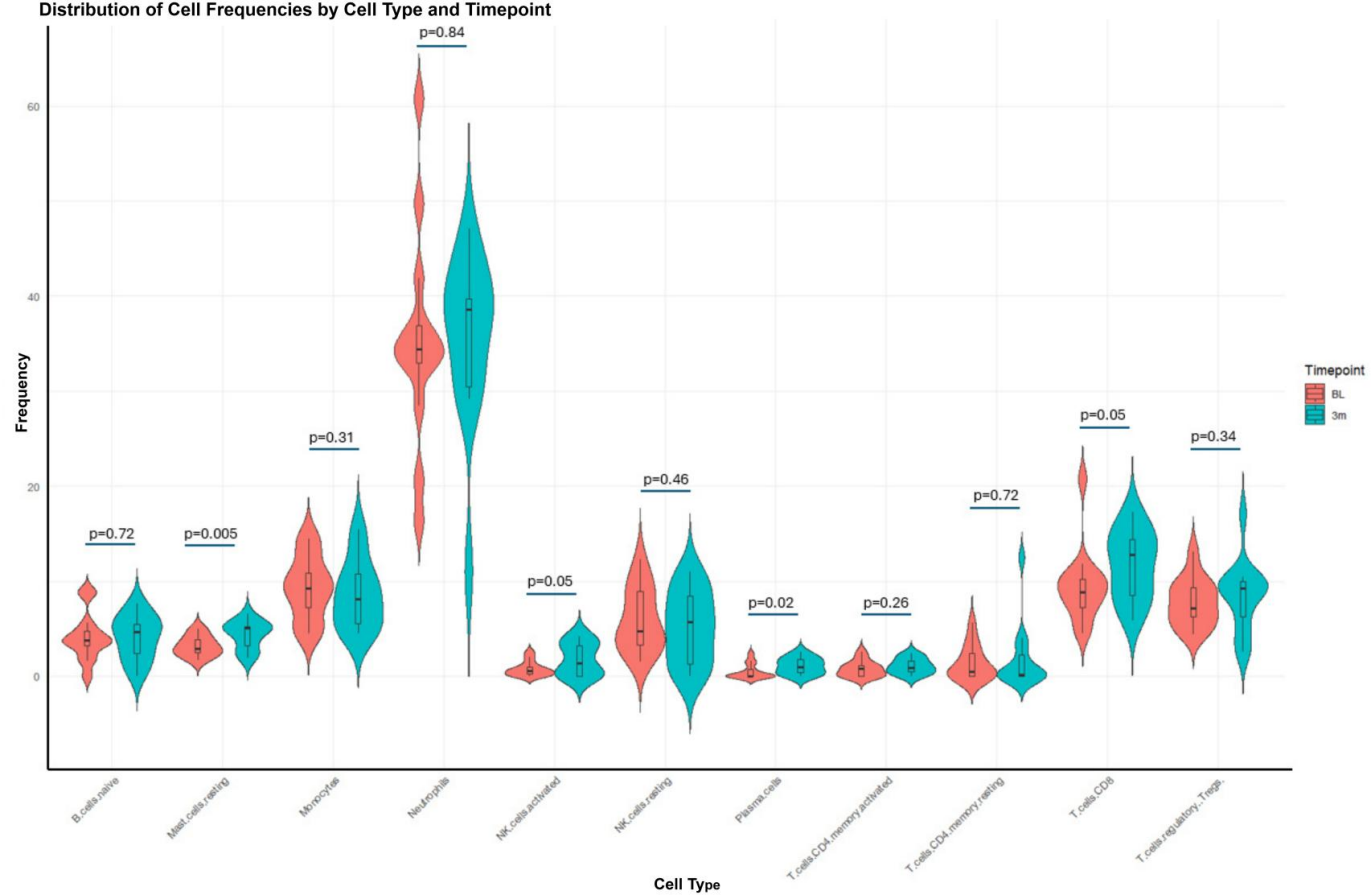
Predicted frequencies of the 11 selected cell types. BL = baseline, 3m = 3months

The cellular landscape in PsA was further characterized by predicting cell-type frequencies within the blood immune cell compartment. Our initial deconvolution output included 22 immune cell types; however, we focused on 11 cell types (naïve B cells, resting mast cells, monocytes, activated NK cells, resting NK cells, neutrophils, plasma cells, activated memory CD4 T cells, resting memory CD4 T cells, CD8 T cells and Tregs) for which frequency estimates were reliably obtained. The remaining cells types were excluded because their frequencies could not be predicted in >50% of the samples at either or both time points, resulting in predominantly zero counts (Supplementary Table S8). Neutrophils dominated the cell populations at both time points {baseline median [interquartile range (IQR)] 34.4% (33–36.9), 3-month median (IQR) 38.6% (30.5–38.6)}, followed by CD8 T cells [baseline median (IQR) 8.8% (7.2–10.2), 3-month median (IQR) 12.8% (8.6–14.4)]. The distribution of the 11 cell types is shown in Fig. 5.

Temporal analysis of the predicted immune cell frequencies between baseline and 3 months revealed significant changes (*P *< 0.05) in several immune cell types, particularly mast cells and plasma cells (Fig. 5), suggesting dynamic modulation of the immune cell landscape after treatment. However, no significant differences were recorded between the response groups at either time point (Supplementary Table S9).

## Discussion

The current treatment pathways for PsA are based on trial and error, with limited understanding of the mechanism of the treatment response. We identified pre-treatment signatures of secukinumab response, some of which were maintained over time. Pathway analyses suggested that perturbations in signalling processes within immune cells may be a mechanistic factor. Further reference-based deconvolution of the whole blood data identified the longitudinal changes in immune cell composition, highlighting the dynamic role of therapy-related immunology.

Differential gene expression analysis between responders and non-responders revealed *IGHV3-64D* and *IGHV1-46* as significantly differentially expressed at both time points, and both showed significant expression changes over the course of treatment. IGHV refers to the variable region of the immunoglobulin heavy chain and is essential for antibody diversity, enabling recognition and binding to a wide range of antigens [28]. Among these, *IGHV4-34* has emerged as a molecular indicator in SLE, with increased frequency correlating with disease severity and complement levels [29]. This study highlights the relevance of *IGHV* transcripts in advancing precision medicine through immunogenetic profiling. In our study, the improved disease activity observed in responders may be attributed to differential expression of *IGHV* transcripts, indicating that B cell repertoire skewing could contribute to the dynamic of the treatment response. Although PsA has traditionally been viewed as a T cell–driven disease, emerging evidence points to a previously underappreciated role for B cells. For instance, following treatment with apremilast, blood-derived B10 cells (IL-10–secreting regulatory B cells) were elevated in PsA patients after being low at baseline and were associated with clinical improvement in both cutaneous and articular symptoms [30]. Similarly, B cell–associated genes were upregulated in response to guselkumab [31]. Elevated immunoglobulin gene expression in PsA skin lesions, potentially driven by POU2F1 activity [32], alongside increased serum autoantibodies targeting LL37 and ADAMTSL5 [33], support a broader immunological role of B cells in PsA. Our findings on the *IGHV* gene further suggest their contribution to both immunoregulation and autoantibody production.

Following IPA, most enriched pathways identified at baseline were immune-related, aligning with the known pathogenesis of PsA [34]. Furthermore, our findings suggested a contribution of metabolic pathways to the therapeutic response of PsA, including creatine metabolism, glycogen degradation, and glycerophospholipid biosynthesis. Inactivation of the creatine transporter SLC6A8 impairs CD8^+^ T cell survival, while *SLC6A8* deletion limits T cell expansion and attenuated TCR-mediated mTORC1 signalling [35]. T cells adopt glycolysis for sustained activation and differentiation, with CD8^+^ memory T cells relying on early glycolytic activation to enable rapid recall responses [36]. Acidic glycerophospholipids regulate TCR signalling by shielding the immunoreceptor tyrosine-based activation motifs (ITAMs) in CD3ɛ and CD3ζ, preventing unintended T cell activation [37]. Upon signalling, Ca^2+^–glycerophospholipid promotes TCR phosphorylation, creating a feedback loop that enhances T cell sensitivity [38]. Although we did not observe enrichment of pathways directly related to the IL-17 axis, the identified metabolic pathways associated with T cell homeostasis suggest an indirect link through immune-metabolic association. Given that IL-17A is produced by T cells, these findings indicate that metabolic activity may influence the therapeutic response to IL-17A activity by modulating T cell function and viability.

Platelet activation emerged as a significantly enriched pathway associated with the therapeutic response, corroborating a previous study by Grivas *et al.* that reported that platelet activation could distinguish between response groups as early as 1 month into treatment [39]. Given their ability to release pro-inflammatory mediators such as cytokines, chemokines, and prostaglandins that enhance immune cell recruitment and vascular permeability [40], platelets may play a more active immunomodulatory role in the therapeutic response. Reports of heightened platelet reactivity [41], and elevated mean platelet volume levels [42] in active PsA further support this finding.

By overlapping WGCNA genes and DEGs, we identified *GMPR, CDC34, DMTN, UBXN6* and *SLC25A* as potential hub genes likely to be important for modulating treatment response. Emerging evidence suggests that *GMPR* may regulate the activity of the Rho-GTPAses pathway [43], proposed as a potential predictor of response to IL-17A inhibitors [11]. In addition, *GMPR* has been identified as one of early responder genes in patients with psoriasis following phototherapy [44]. Aberrations in the E2/CDC34-mediated ubiquitination—NF-κB inflammatory axis, lead to increased production of TNF-α and IL-1β, suppression of synovial cell apoptosis, and consequent synovial tissue proliferation [45, 46]. Dematin (DMTN), actin binding and bundling protein, has been reported to suppress the RhoA signalling pathway [47], which triggers pathological synovial hyperplasia and bone erosion in RA [48]. *UBXN6* enhances JAK-STAT1/2 signalling [49], a key pathway in expanding CD4^+^IL-17A^-^F^+^ and CD4^+^IL-23R^+^ Th17 effector cells in PsA synovial fluid [50]. SLC25A39, a mitochondrial transporter of cytosolic glutathione (GSH), is essential for mitochondrial redox balance. Impaired GSH transport depletes mitochondrial GSH, leading to oxidative stress and mtDNA (mitochondrial DNA) damage. In PsA, mtDNA mutations promote synovial infiltration of T cells and macrophages, elevating TNFα, IL-1β and IFNγ levels [51]. Collectively, these findings reinforce the molecular heterogeneity of PsA and suggest that distinct inflammatory- or immune-associated genes may contribute to variations in therapeutic response. Additionally, they raise important questions about whether a single biomarker or a panel of biomarkers would provide a more accurate and reliable prediction of therapeutic outcomes.

Our baseline phenotypic analysis showed neutrophil dominance in PsA patients, consistent with a previous study [39], suggesting that circulating neutrophilia may reflect general inflammation rather than PsA-specific pathology. In contrast, abundant neutrophils have been observed in PsA-affected tissues, such as the synovium [52] and entheses [53], emphasizing their pathogenic role in local inflammatory lesions. A recent study also reported substantial neutrophil infiltration in the lymph nodes of PsA patients [54].

In our study, deconvolution of blood cell types revealed increased proportions of mast cells and plasma cells in the peripheral blood of PsA patients after 3 months. In contrast, a previous study by O’Byrne *et al.* in PsA synovial tissue demonstrated enrichment with mast cells, whereas plasma cells were relatively scarce [55]. Mast cell degranulation releases IL-17A, which promotes synovial inflammation in SpA and may be a potential mechanism explaining the IL-17A response [56]. No significant differences were observed between responders and non-responders in our study, suggesting a limited role for these circulating cell types as predictive biomarkers.

Our study has several limitations. First, the modest sample size limits statistical power and underscores the need for validation in larger, independent longitudinal cohorts. Second, all participants had established PsA at enrolment, limiting the generalizability of our findings to early PsA patients. Third, the use of outcome measures that primarily assess improvement in peripheral articular manifestations does not capture the therapeutic effects on cutaneous symptoms, despite the known efficacy of secukinumab in treating skin symptoms. Future trials should consider incorporating composite measures that include skin symptoms and physical functioning. Fourth, due to the short duration of this study, it was only possible to assess the predominant initial or primary loss of effectiveness. Finally, although whole-blood transcriptomics is informative and clinically accessible, it may mask cell-type–specific or site-of-disease gene expression and therefore does not fully capture the heterogeneity of the immune cells population involved in the treatment response.

## Conclusion

In summary, we identified baseline predictive biomarkers of therapeutic response, namely *IGHV3-64D* and *IGHV1-46*, through differential expression analysis. Integration of DEGs with WGCNA further revealed five candidate genes potentially involved in modulating treatment outcomes (*GMPR, CDC34, DMTN, UBXN6* and *SLC25A39*). Notably, functional analysis indicated that metabolic pathways may also contribute to shaping the therapeutic response. While these findings require further validation, they uncover the potential contribution of the B cell–mediated immune system, emphasize the marked molecular variability in PsA, and identify dynamics in treatment response pathways, thereby laying the groundwork for predictive biomarker-driven approaches to treatment stratification in PsA.

This study was conducted in accordance with the Declaration of Helsinki, and the OUTPASS protocol was approved by the National Research Ethics Service Committee Northwest—Greater Manchester Central ethics committee (reference 13/NW/0068). All participating patients provided written informed consent.

## Supplementary Material

keag193_Supplementary_Data

## Data Availability

Data are available upon reasonable request.
