# Supplementary material for: Transcriptomic profiling of secukinumab-treated psoriatic arthritis reveals potential novel response-associated pathways
Source: Rheumatology (Oxford). 2026 Apr 15;65(5):keag193. doi: 10.1093/rheumatology/keag193 (PMC13218383; doi:10.1093/rheumatology/keag193)
Supplement: keag193_Supplementary_Data [file keag193_supplementary_data.zip › rhe-25-2884-File008.docx]

**Supplementary Data S1:**

**Members of the Outcome of treatment response in Psoriatic Arthritis Studies Syndicate (OUTPASS)**

Chief investigator: Dr James Bluett

Wrightington, Wigan and Leigh Hospitals NHS Foundation Trust (Dr E G Chelliah (PI), Dr C Chattopadhyay (former PI))

Manchester Royal Infirmary (Manchester University NHS Foundation Trust) (Dr P Ho (PI), Prof. A Barton (former PI), Dr M Castelino, Prof. I Bruce, Dr R Gorodkin, Dr K Hyrich, Dr B Parker)

Salford Royal (Northern Care Alliance NHS Foundation Trust) (Dr H Chinoy (PI), Prof. T O’Neil, Prof. A Herrick, Prof. A Jones, Dr R Cooper, Prof. W Dixon, Dr B Harrison, Dr M Jani, Dr A Low)

Royal United Hospitals Bath NHS Foundation Trust (Dr E Korendowych (PI), Prof. N McHugh, Dr W Tillett)

Aintree University Hospital (Liverpool University Hospitals NHS Foundation Trust) (Dr N Goodson (PI))

East Suffolk and North Essex NHS Foundation Trust (Dr S Lane (PI), Dr L Shand)

Nottingham University Hospitals NHS Trust (Dr I Pande (PI), Dr I Gaywood (former PI), Dr F Rees, Dr M Rutter, Dr S Hayat, Dr J F McHale, Dr A C Jones, Dr P Lanyon, Dr A Gupta, Dr P A Courtney, Dr A Srikanth, Dr A Abhishek)

Royal Devon University Healthcare NHS Foundation Trust (Dr S Kyle (PI), Dr R Manhas)

Mid and South Essex NHS Foundation Trust

Dr A Nandagudi (PI), Dr S Selvan (former PI), Dr A Bharadwaj, Dr N Gendi, Dr R Alshakh

Fairfield Hospital (Northern Care Alliance NHS Foundation Trust) (Dr S Naz (PI), Dr M Ahmad (former PI))

North Manchester General Hospital (Manchester University NHS Foundation Trust) (Dr L Das, Dr M Pattrick, Dr A P Bowden, Dr E E Smith, Dr P Klimiuk, Dr D J Speden)

University Hospitals of Morecambe Bay NHS Foundation Trust (Dr M Bukhari (PI), Dr S Kavaklieva, Dr L Ottewell, Dr M Massarotti)

Midlands Partnership NHS Foundation Trust (Dr J Packham (PI))

Wythenshawe Hospital (Manchester University NHS Foundation Trust) (Dr P Watson (PI), Dr P Sanders (former PI), Dr S Haque, Dr B Pal, Dr E Bruce)

The Mid Yorkshire Hospitals NHS Trust (Dr Z Karim (PI))

Torbay and South Devon NHS Foundation Trust (Dr K Mackay (PI), Dr H Shiels)

Northampton General Hospital NHS Trust (Dr J Taylor (PI), Dr R Jeffery, Dr P Nandi)

Stockport NHS Foundation Trust (Dr C Filer (PI), Dr A Ismail, Dr L Mercer)

North Cumbria Integrated Care NHS Foundation Trust (Dr A Hassan (PI), Dr A Russell)

University Hospitals of Leicester NHS Trust (Dr M Durrani (PI), Dr W Hassan (former PI), Dr A Samanta, Dr P Sheldon, Dr J Francis, Dr A Kinder, Dr R Neame, Dr A Moorthy)

Barts Health NHS Trust (Prof. M Bombardieri (PI), Dr S Kelly (former PI))

Sheffield Teaching Hospitals NHS Foundation Trust (Dr J Maxwell (PI), Dr M Akil, Dr S Till, Dr L Dunkley, Dr R Tattersall, Dr R Kilding, Dr T Tait, Dr K-P Kuet, Dr B Grant, Dr M Kazmi)

St Helens and Knowsley Teaching Hospitals NHS Trust (D Graham (PI), Dr V E Abernethy, Dr A R Clewes, Dr J K Dawson)

NHS Greater Glasgow and Clyde (Dr S Siebert (PI), Dr G Fragoulis)

Royal Liverpool and Broadgreen (Liverpool University Hospitals NHS Foundation Trust) (Dr D Mewar (PI), Dr E J Tunn, Dr K Nelson, Dr T D Kennedy, Dr C Dubois)

The Dudley Group NHS Foundation Trust (Dr K Douglas (PI), Dr E Ladoyanni, Dr C Koutsianas, Dr N Erb, Dr R Klocke, Dr A J Whallett, Dr A Pace, Dr R Sandhu, Dr H John)

Portsmouth Hospitals University NHS Trust (Dr S A Young Min (PI), Dr A Cooper, Dr J M Ledingham, Dr R G Hull, Dr F McCrae, Dr Wong, Dr Shaban)

Mid Cheshire Hospitals NHS Foundation Trust (Dr K Putchakayala (PI))

The Rotherham NHS Foundation Trust (Dr R Kumari (PI), Dr G Smith (former PI))

South Warwickshire NHS Foundation Trust (Dr C Marguerie (PI))

Homerton Healthcare NHS Foundation Trust (Dr P Reynolds (PI), Dr C Thornton (former PI), Dr C Gorman, Dr C Murphy)

Tameside and Glossop Integrated Care NHS Foundation Trust (Dr D Roy (PI))

Lancashire and South Cumbria NHS Foundation Trust (Dr S Horton (PI))

University College London Hospitals NHS Foundation Trust (Dr M Castelino (PI))
